# The complete chloroplast genome sequence of *Melliodendron xylocarpum* (Styracaceae)

**DOI:** 10.1080/23802359.2019.1677521

**Published:** 2019-10-21

**Authors:** Zixun Zhao, Xiaogang Xu, Lili Tong, Yaoqin Zhang, Yabo Wang

**Affiliations:** a College of Biology and Environment, Nanjing Forestry University, Nanjing, China;; b Co-Innovation Center for Sustainable Forestry in Southern China, Nanjing Forestry University, Nanjing, China;; c School of Horticulture & Landscape Architecture, Jinling Institute of Technology, Nanjing, China

**Keywords:** Phylogenomics, Styracaceae family, complete chloroplast genome

## Abstract

*Melliodendron xylocarpum* Handel-Mazzetti is a rare tree, distinct member of the family Styracaceae. In this study, we determined the complete chloroplast (cp) genome sequence of *M. xylocarpum* in an effort to provide genomic resources useful for promoting its conservation. The entire cp genome was determined to be 157,165 bp in length. It contains the typical structure and gene content of angiosperm plastome, The plastome contains a large single-copy (LSC) and a small single-copy (SSC) regions of 90,193 and 18,486 bp, respectively, which were separated by a pair of 24,243 bp inverted repeat (IR) regions. The genome contained 130 genes, including 85 protein-coding genes, 37 tRNA genes, and 8 rRNA genes. The GC content of *M. xylocarpum* genome is 37.21%. The complete plastome sequence of *M. xylocarpum* will provide a useful resource for the conservation genetics of this species as well as for the phylogenetic studies for Styracaceae. Phylogenetic analysis revealed that *M. xylocarpum* is closely related to *Changiostyrax dolichocarpus* H.S.Lo & D.Fang, but forms an independent evolutionary branch.

*Melliodendron xylocarpum* Handel-Mazzetti, one of the rare Chinese trees, is a broad-leaved tree species with beautiful flowers. *Melliodendron xylocarpum* is distributed southern China which has high medicinal value and can be as applied for ornamental purposes. Despite this progress in understanding Styracaceae systematics, many of the relationships among genera in the family remain poorly resolved. Comparison of complete plastome sequences further provides the opportunity to explore sequence variation and molecular evolutionary patterns associated with gene loss, rearrangements, duplication, and transfer events (Walker et al. 2014; Weng et al. 2014). Here, we characterised the complete cp genome sequence of *M. xylocarpum* (GeneBank accession number: MN175625) based on Illumina pair-end sequencing to provide a valuable complete cp genomic resource.

The total genomic DNA was extracted from the fresh leaves of *M. xylocarpum* grown in Qixia Mountain (N 32.1566, E 118.9690) in Nanjing, Jiangsu, China. The voucher specimen was kept in the herbarium of Nanjing Forestry University (accession number: NF2018689). The whole genome sequencing was conducted by Nanjing Genepioneer Biotechnologies Inc. (Nanjing, China) on the Illumina Hiseq platform. The raw reads were filtered by CLC Genomics Workbench v9, and the obtained clean reads were assembled into chloroplast genome using SPAdes(Bankevich et al. 2012). Finally, gene structure annotation was carried out with CpGAVAS (Liu et al. 2012) and the physical map was generated with OGDRAW (Lohse et al. 2013). A phylogenetic tree was infered based on the Maximum Likelihood (ML) by using MAFFT (Katoh and Standley 2013).

The plastome of *M. xylocarpum* was determined to comprise double-stranded, circular DNA of 157,165 bp containing two inverted repeat (IR) regions of 24,243 bp each, separated by large single-copy (LSC) and small single-copy (SSC) regions of 90,193 and 18,486 bp, respectively. The genome contained 130 genes, including 85 protein-coding genes, 37 tRNA genes, and 8 rRNA genes. Five protein-coding genes, seven tRNA genes, and four rRNA genes were totally duplicated in IR region. Twelve genes contained one intron and two genes (*clpP* and *rps12*) contained two introns. The overall GC content of *M. xylocarpum* cp genome is 37.21% and the corresponding values in LSC, SSC, and IR regions are 35.34%, 30.57%, and 43.23%, respectively.

To ascertain the phylogenetic evolution of *M. xylocarpum*, the fasta format file containing all the chloroplast genome sequences of 25 species (20 Styracaceae chloroplast genomes and 5 taxa as outgroup). The results of phylogenetic analysis show that *M. xylocarpum* forms a separate branch when compared with other plants in this study, which is more closely related to *Changiostyrax dolichocarpus* (Figure 1). The complete plastome sequence of *M. xylocarpum* provides an important data set for the conservation genetics of this species.

**Figure 1.**
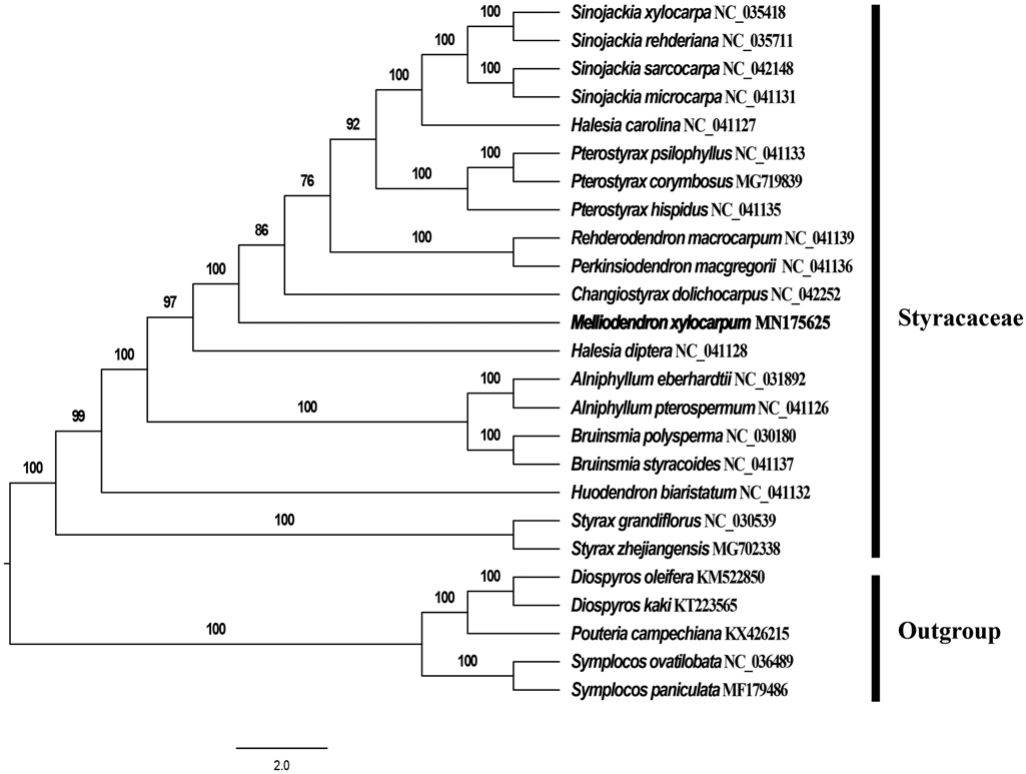
Phylogenetic tree inferred by maximum-likelihood (ML) method based on the complete chloroplast genome of 25 representative species. The bootstrap support values are shown at the branches.
